# Restructuring Crisis Response Programs as Civilian-Led: A Scoping Review

**DOI:** 10.1007/s10488-026-01514-w

**Published:** 2026-06-29

**Authors:** Megan W. Rowe, Allannah Nguyen, Leyna Lowe, Kara Fletcher, Andrew D. Eaton

**Affiliations:** 1 Faculty of Health, Dalhousie University, Halifax, Canada; 2 Faculty of Social Work, University of Regina, Regina, Canada; 3 Canadian Mental Health Association, Ottawa, Canada; 4 Jane Addams College of Social Work, University of Illinois at Chicago, Chicago, USA; 5 Factor-Inwentash Faculty of Social Work, University of Toronto, Toronto, Canada

**Keywords:** Civilian-led crisis response, Non-police crisis response, Alternative crisis response teams, Mental health

## Abstract

Civilian-led crisis response teams provide emergency mental healthcare for individuals in acute distress in community settings, often without the involvement of police as first responders. These models represent one of several emerging alternatives to police-led crisis response and have gained attention for their potential to improve safety, trust, and care outcomes. Reports included in this scoping review discussed: the development, need, potential, implementation, and outcomes of civilian-led crisis response models. Covidence software was utilized by two independent reviewers to search 11 databases, with a third reviewer resolving conflicts. A dataset of 46 reports were then analyzed with thematic content analysis by a multidisciplinary team using critical theories to offer an exploration of how civilian-led crisis teams have begun to address the harms of policing responses to mental health. In exploring the key processes for civilian-led crisis response teams, three themes emerged. The first theme, *Decentering Police,* explores the growing collective awareness of the harms associated with police-involved crisis intervention and the corresponding need for alternative approaches, alongside efforts to establish a team composition that is intentionally distinct. The second theme, *Team Scope of Practice,* explores the subthemes of dispatch logistics and defining criteria for response. *Team Sustainability* is the third theme and explores how social and political will shape the uptake and long-term operation of civilian-led crisis programs.

## Introduction

The involvement of police in responding to crisis situations, referred to as policing mental health (Koziarski, 2018), has drawn increasing scrutiny (Haag, 2022; Marcoux & Nicholson, 2018). A mental health crisis is broadly understood as “emotional distress that exceeds an individual’s coping mechanisms” (Roennfeldt et al., 2024, p. 2). These incidents frequently lead to family, friends, and bystanders placing emergency calls, which traditionally get dispatched to police as the primary responders to crisis situations (Jackson & Bradford, 2019; Pepler & Barber, 2021). Individuals experiencing a mental health crisis face disproportionate contact with police (Pope et al., 2023), and while individuals experiencing emotional distress are more likely to be subject to violence than perpetrate it (Black & Calhoun, 2022; Cotton & Coleman, 2010; Shore, 2015), the biases that underpin policing mental health often lead police to approach crisis situations with the expectation of violence (Shore, 2015).

Crisis response is shaped by more than emergency protocols; it reflects the structural and institutional conditions that determine how distress, risk, and responsibility are understood (Foucault, 1961; Maynard, 2017; Nishar et al., 2025; Thompson et al., 2018). Over time, austerity measures and neoliberal policy priorities have limited investment in social infrastructure (Brown et al., 2022; Harvey, 2005; Mukherjee, 2022; Shimrat, 2013; Walby, 2022). Community-based models of care have therefore not been fully realized, contributing to an expanded role for law enforcement (Shimrat, 2013; Walby, 2022). In the absence of accessible, well-resourced services, police have increasingly become the default responders to crisis situations (Brown et al., 2022; Mukherjee, 2022).

At the same time, police-led crisis intervention is shaped by interpretive logics that extend beyond service gaps, with significant implications for access to mental health care (Marcus & Stergiopoulos, 2022; Nishar et al., 2025). Critical and interdisciplinary scholarship shows that understandings of distress and risk are influenced by sanist, racist, and colonial assumptions that shape how care is delivered by institutions (Liegghio, 2013; Marcus & Stergiopoulos, 2022; Meerai et al., 2016; Yee, 2022) and experienced by people seeking support (Nishar et al., 2025). Sanism, a central concept within mad studies, refers to the systemic marginalization of people perceived as mentally ill (Herson, 2018; Poole et al., 2012). Sanism upholds assumptions about mental health crises that conflate distress with violence or dangerousness (Johnstone & Boyle, 2018). Intersecting with racism and settler colonialism, it produces distinctly anti-Black and anti-Indigenous patterns of interpretation, wherein expressions of distress by Black and Indigenous people are more likely to be read as threatening (Benjamin, 2003; Hon-Sing Wong, 2016; Maynard, 2017; Simpson, 2017). As Meerai et al. (2016) note, “sanism exists on a continuum depending on privilege, and it is always and especially compounded when it is visited on racialized bodies” (p. 22).

Systemic biases influence how psychiatrized (Hon-Sing Wong, 2024; Lee, 2013; Liegghio, 2013) and racialized individuals (Hon-Sing Wong et al., 2022; Maynard, 2017; Meerai et al., 2016) are perceived and treated during moments of crisis. For example, data from a Canadian database **documenting** police-involved fatalities between 2000 and 2017 **indicate** that 68% (n = 377) of those killed were experiencing acute distress, with Black individuals comprising 8.63% of deaths despite representing 2.92% of the population, and Indigenous individuals accounting for 16% of deaths despite comprising 4.21% of the population (Marcoux & Nicholson, 2018; Singh, 2020). In response, efforts to improve crisis response have emerged within existing law enforcement structures, producing two dominant models: Crisis Intervention Teams (CITs) and co-response (Redgate et al., 2025). CIT training seeks to equipt police officers to recognize and de-escalate distress while facilitating service referrals, yet research shows limited impact of these models on arrests, use-of-force rates, or fatalities (Marcus & Stergiopoulos, 2022; Rogers et al., 2019; Shadravan et al., 2021; Taheri, 2016). Co-responder models pair police with mental health clinicians to bridge perceived gaps between health and law enforcement systems (Iacobucci, 2014; Rosenbaum, 2010). However, because police remain central to decision-making and risk assessment**,** these models may continue to situate emotional distress within criminal legal frameworks rather than offering a fundamentally different response (Eaton et al., 2025; Marcus & Stergiopoulos, 2022; Pepler & Barber, 2021). A recent randomized, controlled trial found no significant differences in effectiveness between co-responder teams and traditional police responses (Lowder et al., 2024). Importantly, neither CIT models nor co-responder teams constitute a non-police alternative.

Traditional mobile crisis teams, staffed by mental health clinicians such as psychiatrists, nurses, social workers, and/or psychologists, have long formed part of the broader mental health crisis response ecosystem (Zealberg et al., 1993). Emerging alongside deinstitutionalization and community psychiatry initiatives, these teams were initially embedded within hospitals, community mental health centres, and crisis services to provide rapid crisis assessment, de-escalation, and linkage to care within community settings and outside of emergency departments (Geller et al., 1995; Goldman et al., 2023; Hoult, 1996). Mobile crisis services have historically focused on reducing unnecessary hospitalization and emergency department use while increasing engagement in community-based care (Goldman et al., 2023). Today, mobile crisis teams continue to operate within broader behavioral health systems and increasingly form part of coordinated crisis continuums linked to crisis lines such as 988 and, in some jurisdictions, mental health-related 911 diversion pathways (Goldman et al., 2023). At the same time, mobile crisis teams remain operationally diverse across jurisdictions, with varying staffing structures, dispatch pathways, and relationships with law enforcement (Goldman et al., 2023).

Civilian-led crisis response teams have emerged as a distinct model, designed to provide community-based support without police involvement. These programs reflect broader efforts to decouple distress from automatic associations with danger and to reconceptualize crisis as a health and social matter rather than a public safety threat (Haag, 2022). Civilian-led teams have been piloted or implemented across multiple jurisdictions, including the United States (Townsend et al., 2023), Canada (Egan-Elliott, 2021), Australia (Justice Action, 2023), and Norway (Karlsson et al., 2012). Although interest in these models is growing, the research base remains limited; for instance, a recent rapid review identified limited high-quality research examining non-police crisis responses models (Marcus & Stergiopoulos, 2022). In response, the current research offers an updated exploration of the restructuring of crisis response models into civilian-led teams: teams that remove police from frontline intervention and instead rely on trained, unarmed personnel such as mental health practitioners, crisis workers, and/or peers (DeLaus, 2020). Drawing on critical social theories, we consider these models as operational alternatives and developments that reflect shifting understandings of care, safety, and decision-making in moments of crisis.

Our analysis is theoretically informed by mad studies, the Power Threat Meaning Framework (PTMF), and critical race theory (CRT). Mad studies challenges biomedical and policing paradigms by conceptualizing distress as a meaningful response to lived experience, often rooted in structural violence, trauma, or marginalization (Poole et al., 2012). The PTMF complements this orientation by emphasizing questions such as “what has happened” and by foregrounding collaborative meaning-making and contextual forms of support (Johnstone & Boyle, 2018). CRT extends this foundation by tracing how the criminalization of distress is intertwined with racism and Western colonialism, shaping whose behaviours are read as dangerous and whose distress is taken up as legitimate (Badwall, 2022; Maynard, 2017; Page & Woodland, 2023). These frameworks offer an integrative lens for understanding how civilian-led teams emerge within, resist, and reconfigure existing crisis response systems.

## Methods

A scoping review was employed to comprehensively map and synthesize existing knowledge on civilian-led crisis response teams, ensuring a broad exploration of the available literature. The research questions guiding this review was “How have crisis mental health response systems (i.e., program models) been restructured into civilian-led teams to minimize crises, lessen or mitigate police presence, and redirect people experiencing crisis mental health events from emergency room visits to more sustainable care pathways?.” Here, “more sustainable care pathways” refers to forms of crisis support that move beyond repeated emergency interventions by providing on-site support, de-escalation, and connection to community-based services outside of emergency departments. In addressing this, we explore: What are the key processes (e.g., legislation, community-based partnership) that facilitate these restructurings of crisis mental health response systems?

### Search Procedure

Following scoping review guidelines (Munn et al., 2018; Pham et al., 2014) we registered the review with Open Science Framework and conducted scholarly database searches alongside searching relevant professional networks and works in progress. We identified relevant studies by completing 11 database searches that included APA PsychInfo, APA PsycArticles, CINAHL & MEDLINE Combined Search, Criminal Justice Database (collection and abstracts), CQ Researcher, ProQuest Health & Medical Collection, ProQuest Nursing & Allied Health Database, SAGE Journals, Sociological Collection (Sociological Abstracts), Social Work Abstracts, and ProQuest Dissertations and Theses. We included grey literature, media, and reports. Key terms used to conduct the searches included “non police” AND [“mental health” or “crisis” or “emotional distress”] AND [“community crisis response” OR “non police crisis response”] AND [alternative mental health crisis response]. We also conducted manual searches to identify literature that might have been missed within our search of scholarly databases. Complete search syntax is available on Borealis (Rowe & Eaton, 2025).

### Inclusion Criteria

A report was included if it examined a civilian-led crisis response team and focused on acute distress (or "mental health") specific crisis intervention. For this research, a civilian response model is defined as a crisis response approach involving trained mental health response workers, including but not limited to mental health clinicians such as social workers, psychologists, nurses, and/or trained peers, with no sworn members (primarily police officers and occasionally paramedics). Paramedics may serve as sworn members, particularly in integrated public safety departments where personnel are cross-trained to perform multiple roles (Hilal & Jones, 2014).

To be included, the literature needed to recognize or explore at least one of the following dimensions:

- The need for non-police crisis response models
- The potential of these models
- Their development
- Their implementation
- Outcomes associated with their use
- Their impact on crisis response
- The efficacy of such models

Additionally, studies were required to discuss programs that serve the public and act as the first point of contact during crisis situations. We included empirical research and grey literature. Only English-language literature published from the year 1999 onward was considered.

### Screening

**Fig. 1:**
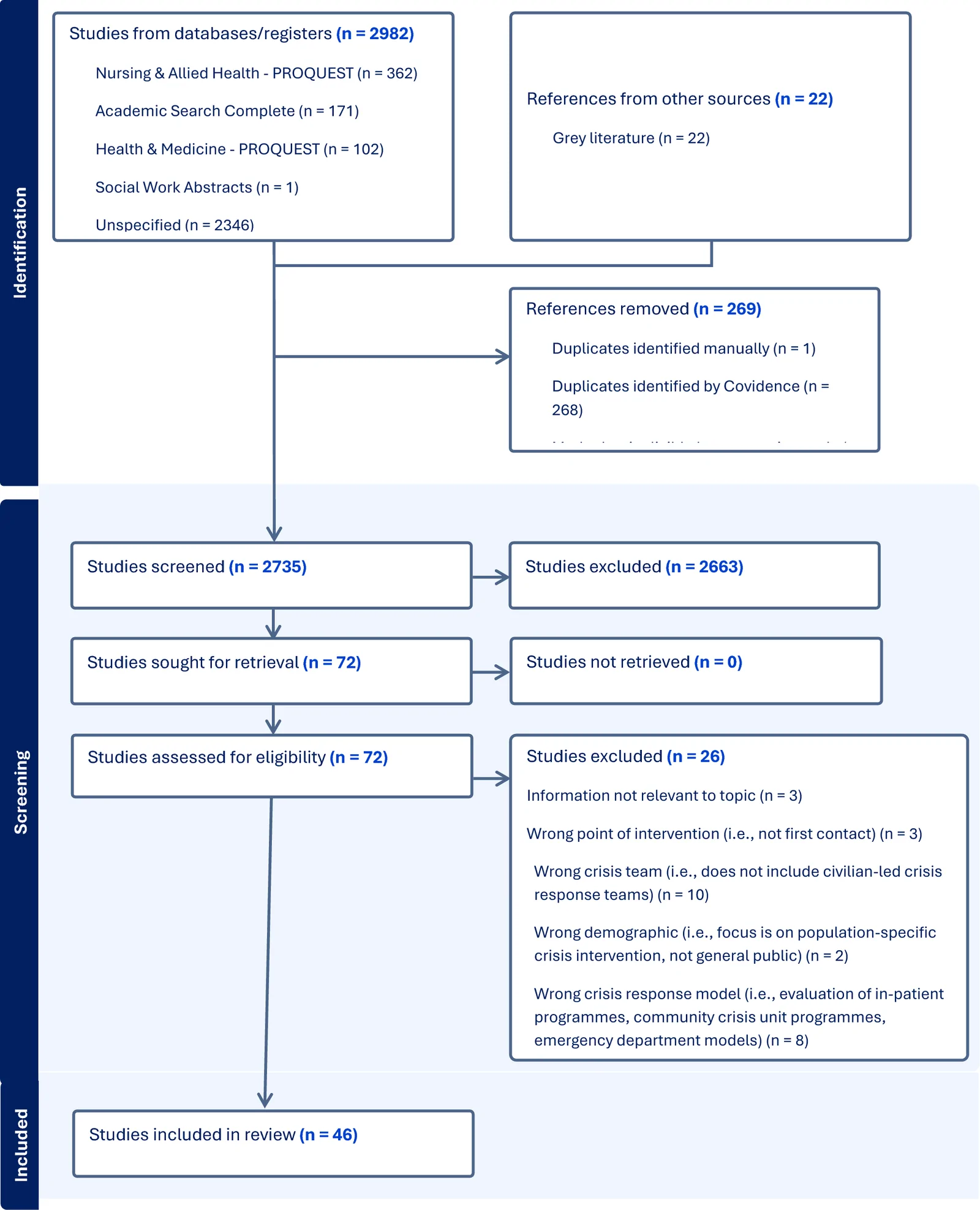
PRISMA chart

Screening was conducted using Covidence software by two independent reviewers following the established eligibility criteria. Upon completion, the reviewers met to discuss the screening results, and a third reviewer was involved to resolve screening discrepancies where needed. Subsequently, each reviewer was assigned articles to complete a full-text screen. In total, 46 articles were deemed eligible for inclusion. Refer to Fig. 1 for the PRISMA flow chart.

### Data Extraction and Analysis

Data extraction and analysis was completed by four trained research assistants, extracting data pertaining to team composition, funding, and dispatch of team (i.e., 911, 211), amongst other key data points. The extraction spreadsheet is available on Borealis (Rowe & Eaton, 2025). Thematic content analysis was completed by eight independent coders, each of whom was either someone working within crisis response, a student in a health-related field, a policy analyst, a mental health advocate, and/or someone who brings with them lived experience of psychological distress. Each coder was assigned 5–6 articles and provided with detailed coding guidelines (see supplementary file) to ensure consistency in their analysis. NVIVO 12 software was used to systematically organize and analyze the data. Coders independently reviewed and coded their assigned articles, after which the team met to discuss findings, resolve discrepancies, and refine the thematic framework. The first and last authors drafted themes and subthemes based on the team meeting and all authors approved the results as presented herein.

### Quality

During the screening stage, it was identified that most literature came from grey or media sources. Accordingly, the Mixed Methods Appraisal Tool (MMAT) Version 2018 (Hong et al., 2018) was not an appropriate tool for evaluation. Instead, an adapted version of the AACODS (Authority, Accuracy, Coverage, Objectivity, Date, Significance) tool (Tyndall, 2010) was used. Originally developed to assess grey literature, the AACODS framework was modified for this scoping review to ensure its applicability to both grey and media sources. The adapted tool included 10 questions to evaluate authority and source credibility, corroboration of information, topic coverage, objectivity, and publication date (see supplementary file). Each question was dummy-coded: a 1 was given if the response to the criteria was a “Yes” or a 0 if the response was a “No” or “Can’t Tell.”, with a total possible score of 10. Articles were given scores ranging from 0% (low quality) to 100% (high quality). Appraisal was conducted independently by one reviewer. A second reviewer cross-checked their work, making appropriate changes where necessary, such as determining publication source and/or clarifying publication date. The appraisal results are posted on Borealis (Rowe & Eaton, 2025).

## Results

A total of 46 articles were included in this scoping review, the majority of which were grey literature (n = 22) and media reports (n = 21). The included articles ultimately ranged from 1999 to 2024, though the literature was concentrated in more recent years, with all included sources published between 2017 and 2024 except for a single article from 1999. Additionally, the review incorporated two qualitative reviews and one descriptive quantitative study. An adapted version of the AACODS tool was used to appraise the quality of each included article. Twenty-three articles received a score of 100%, five articles received a score of 90%, eight articles received a score of 80%, eight articles received a score of 70%, and two articles received a score of 60%.

**Fig. 2:**
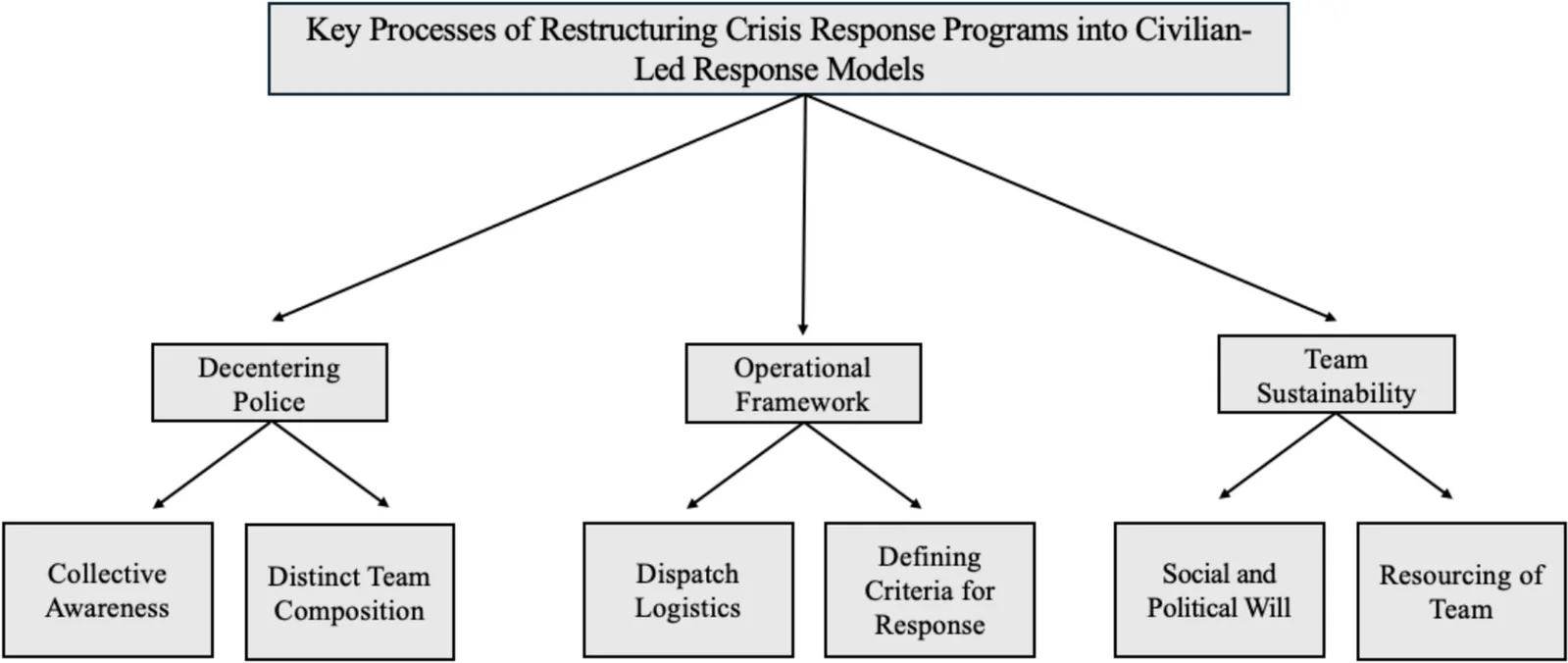
Thematic analysis

Three primary themes were identified regarding key processes involved in restructuring crisis response models into civilian-led teams, each with corresponding subthemes. The first theme, *Decentering Police*, captures the role of collective awareness and the importance of establishing a distinct team composition. The second theme, *Team Scope of Practice*, addresses considerations related to dispatch number and defining criteria for response. The third theme, *Team Sustainability*, highlights the influence of interest holder perceptions and political will, as well as the need for adequate resourcing. Figure 2 illustrates these thematic findings.

### Theme 1: Decentering Police

Thirty-nine articles discussed the decentering of police within crisis response as a central component to structuring civilian-led crisis response services. The theme of decentering police consists of two subthemes: collective awareness of the harms policing mental health and establishing a distinct team composition.

#### Sub-Theme 1.1: Collective Awareness

Twenty-seven articles identify collective awareness of the harms of policing mental health – including the recognition that police involvement in crisis intervention often escalates situations (e.g., Beck & Stagoff-Belfort, 2022; Reach Out Response Network, 2020) and disproportionately harms racialized communities (e.g., Anene et al., 2023; Canady, 2021) – as a key driver in the shift toward establishing civilian-led crisis response teams in Norway, Canada, Australia, and the United States. Racialized communities and individuals with lived experience of acute distress or crisis have long advocated against the disproportionate policing they face and the resulting harms, including fatal outcomes (Reach Out Response Network, 2020). The literature suggests that calls to reimagine public safety by reducing police involvement in healthcare – particularly in crisis response – are driven in part by the argument that situations requiring emotional support and de-escalation should not be met with a police response (Karlsson et al., 2012) as law enforcement “are not trained or equipped to handle [crisis intervention] appropriately” (Townsend et al., 2023). The literature describes a consciousness-raising process in which longstanding advocacy efforts gained broader recognition in public and political discourse following highly publicized incidents of police violence that intensified public concern of policing and crisis intervention practices. In jurisdictions that have implemented civilian-led crisis teams, increasing awareness of the disproportionately harmful consequences of police interactions – particularly for Black, Indigenous, and individuals experiencing acute distress – has further reinforced the urgency of these efforts (Legal Defense Fund & Bazelon Center, 2022).

Anene and colleagues (2023), Canady (2021), van Lier (2021, 2022), Yousif (2020), Local Progress Impact Lab (2022), amongst others, cite the tragic murder of George Floyd by police officer Derick Chauvin in May 2020 in Minneapolis, U.S.A., as a catalyst event that precipitated calls for police reform and abolition. This event caused the public to question the legitimacy of policing more broadly and its involvement in crisis intervention more specifically, making longstanding demands for systemic change more difficult for policymakers to ignore. As Watson and El-Sabawi (2023) explain, it was in the wake of George Floyd’s murder that “policymakers have begun to take seriously demands to…provide the public with alternatives to police when they are in need of help” (p. 1). Similarly, van Lier (2021) notes that initiatives to explore non-police crisis response models gained traction as high-profile killings of Black people by police have “vaulted the actions of law enforcement” (p. 1) into the public eye.

While George Floyd’s murder exposed the deadly realities of police violence, the death of Regis Korchinski-Paquet during a crisis intervention highlighted the specific dangers of police involvement in crisis response, further building momentum for systemic transformation. Chambers and Deshman (2024), CityNews Staff (2021), Ngabo (2021), and Yousif (2022a, 2022b) highlight the death of Regis Korchinski-Paquet during a police-led crisis intervention in Toronto in 2020 as pivotal in advancing the push for non-police crisis response services, directly contributing to the development of the Toronto Community Crisis Service. In sum, sustained collective mobilization, particularly through pressure on elected officials, played a crucial role in advancing these discussions and prompting key decision-makers to consider alternative crisis response models.

#### Sub-Theme 1.2: Establishing a Distinct Team Composition

All literature included in this scoping review emphasized the importance of establishing a distinct team composition when restructuring crisis response programs into civilian-led models, particularly by removing and excluding police from these teams to provide safer, more appropriate care to individuals experiencing acute distress. While the literature suggests civilian-led crisis teams vary in their staffing and team composition (e.g., Karlsson et al., 2012), a consistent defining feature across models is the deliberate exclusion of police from team staffing. Civilian-led models recognize the inherent harm introduced by police presence in any form and have responded by eliminating police from team composition. In addition to this fundamental distinction, these teams also stand out for their intentional inclusion of ‘peers,’ referring to individuals who bring with them the knowledge and wisdom of living or lived experiences of mental health challenges and/or experiences of acute distress (Mental Health Commission of Canada, 2025). As per Wyton (2022), “the comfort and non-judgement from peers is so sacred and so needed," (p. 2).

Beyond the exclusion of police, some civilian-led crisis teams also distinguish themselves through an emphasis on voluntary, consent-based intervention, recognizing the inherent power imbalances that can occur within crisis and clinical encounters. Specifically, Chambers and Deshman (2024) and Provincial System Support Program & Makwa (2023) discuss concerns regarding non-consensual approaches to care, including involuntary assessment or hospitalization, which may be experienced as coercive by individuals in crisis. Further, these dynamics can mirror some of the distress associated with police involvement (Chambers & Deshman, 2024). Clinicians may nonetheless be legally and professionally required to initiate these interventions in situations involving perceived risk or safety concerns. These considerations encouraged some models to lean more heavily on peer-led and/or Indigenous- and Black-led team composition, noting:

This approach recognizes the barriers to service that are inherently connected to power imbalances between the person providing care and their client… seeks to achieve better care through being aware of difference, decolonizing, considering power relationships, implementing reflective practice, and by allowing the patient to determine whether a clinical encounter is safe” (Provincial System Support Program & Makwa, 2023, p.43).

The United States’ (US) Substance Abuse and Mental Health Services Administration’s (SAMHSA) *2020 National Guidelines for Behavioral Health Crisis Care* emphasize non-police crisis response teams as best practice for mental health crisis intervention and are heavily cited as the evidence base to support the legitimacy of civilian crisis teams, including ones outside of the US (Canady, 2021; Karlsson et al., 2012; Ryder & Nash, 2024). These guidelines outline that a public health approach rather than a law enforcement response should be the foundation for crisis care. Gillis (2023) notes “we hope that by removing police, we’ll reduce stigma, and people will be able to see that [emotional distress] isn’t a crime, it’s a health crisis” (p.6). Beck and colleagues (2022) outline that police involvement often escalates emotional distress, resulting in harm towards the individual in crisis. This is particularly true for Black and other non-white people; “Black people, in contrast to most white people, live with the fear that police will hurt them or their family members” (Beck & Stagoff-Belfort, 2022, p. 2). In an anonymous online survey conducted across Nova Scotia, Canada, participants who self-identified as having lived experience with mental health or substance use challenges reported low trust in police when responding to crises (Livingston & Chambers, 2024). The findings also highlighted that non-police, civilian-led crisis teams were widely perceived as “beneficial, comfortable, helpful, appropriate, and preferred” (Livingston & Chambers, 2024, p. 4).

The importance of developing civilian-led programs in collaboration with community members to meet the needs and priorities of those most impacted was equally emphasized (e.g., CSG Justice Center, 2021; Spolum et al., 2023; Yousif, 2020). The Local Progress Impact Lab (2022) notes “community engagement that centers the voices and input of those most likely to be served by [civilian-led programs] is critical” (p. 18), while Reach Out Response Network’s Asante Haughton cited by Yousif (2020) expresses the importance of “prioritizing the community voice [such that] whatever is built is representing what folks are asking for” (p. 2). Nonko (2020) explains that a civilian-led pilot program in California was informed by “family members of individuals killed by police, as well as experts in non-police responses to crises” (p. 1).

Chrastil (2023), Yousif (2020), and van Lier (2022) each explain that within large cities that have implemented civilian-led models (e.g., Toronto, New Orleans, Cleveland), a task force was first implemented to consult and engage community members and other key interest holders, ensuring their insights were involved in program design and development. In Toronto, Canada, specific attention was placed on Black and Indigenous-led engagement in initial development process for the Toronto Community Crisis Service (Yousif, 2020).

Within this scoping review’s included literature, civilian-led teams were described as consisting of: nurses paired with mental health workers including clinicians and/or peers (Kearnes, 2022; Mental Health Weekly, 2022a, 2022b; Yousif, 2022a, 2022b, 2023), mental health workers (Reach Out Response Network, 2020; Townsend et al., 2023), behavioral health crises workers and licensed clinicians (Ryder & Nash, 2024; Towles, 2021a, 2021b), trained crisis responder volunteers (Nonko, 2020; Wellborn, 1999), and most commonly, mental health professionals and peers (Campbell, 2023; CityNews Staff, 2021; Fleming, 2021; Provincial System Support Program & Makwa, 2023; Towles, 2021a, 2021b; van Lier, 2022, Wyton, 2022; Ziafati, 2022).

### Theme 2: Operational Framework

Thirty-two of the included literature sources discussed the overarching role of determining an operational framework in the process of restricting crisis response models into civilian-led teams. Particularly, navigating dispatch logistics and the defining criteria for a response emerged across the literature as critical components of establishing a team’s operational framework.

#### Sub-Theme 2.1: Dispatch Logistics

Determining dispatch logistics, including the number by which a team can be contacted as well as its hours of operation, was discussed in 25 articles. The literature points to deliberations regarding the point of access for a team as well as the hours of operation that a service is available, indicating there is no universal standard. The most common dispatch points indicated throughout the literature include the phone numbers 911, 211, and 988. The dispatch procedure consistently described across the literature included the initial calls for service, subsequent screening by the call-taker, and finally dispatch of the team.

Some articles argue that using 911 as the dispatch number for civilian-led crisis response teams is crucial for seamless integration with emergency services such as police, fire, and EMS (e.g., Ryder & Nash, 2024; Yousif, 2020, 2022a). Additionally, 911 is widely considered the most accessible option, as it is a universal emergency number that can be dialed free of charge from cell phones or payphones, even without a working SIM card (Yousif, 2020). For calls made to 911, some sources describe a transfer process in which calls are first screened by a 211 operator before the appropriate response team is dispatched (e.g., Chrastil, 2023; CityNews Staff, 2021). Others propose direct integration, allowing 911 operators to dispatch civilian-led teams alongside traditional emergency responders (e.g., CSG Justice Center, 2021). However, some of the literature raised concerns about linking crisis response teams to 911, cautioning that alternative contact points may feel safer and more trustworthy, particularly for communities that do not associate 911 with safety (e.g., Beck & Stagoff-Belfort, 2022; Mellins, 2021; Mental Health Weekly, 2022a, 2022b; Townsend et al., 2023). A hybrid approach was also discussed in the literature, suggesting multiple access points – such as being able to access teams by calling 911, 211, or a dedicated crisis team number – to ensure broader accessibility and choice for those in crisis (e.g., Chrastil, 2023; Reach Out Response Network; Yousif, 2022a, 2022b).

The literature demonstrates variations regarding the hours of operation of civilian-led crisis response services. While some programs were described as in operation 24/7/365 (e.g., Karlsson et al., 2012; Kearnes, 2022; Reach Out Response Network, 2020), others were limited in their hours and days of operation, noting the primary barriers to offering the service 24/7/365 included limitations in funding and securing staff (e.g., Wyton, 2022). Notwithstanding, across all models discussed, the goal is to “to offer rapid assessment with 24/7 availability” (Karlsson et al., 2012, p. 3).

#### Sub-Theme 2.2: Defining Criteria for a Response

The literature (n = 37) examined the types of situations that civilian-led crisis teams are designed to respond to. This "scope of response" refers to the specific kinds of situations the teams are equipped to handle. Typically, these teams are dispatched when a caller uses certain key words or phrases that match the team’s predefined criteria for intervention. Across the literature, the most common keywords used to delineate the types of situations these teams respond to include ***‘mental health crisis’ and ‘substance use crisis’*** (Fagan et al., 2025; Ziafati, 2022), ‘*non-emergency crisis’* (e.g., CSG Justice Center, 2021; Kearnes, 2022), and ‘*non-violent incidents’* (e.g., Egan-Elliott, 2021; Provincial System Support Program & Makwa, 2023; van Lier, 2022). Operators are trained to assess incoming emergency calls and identify those that meet the established criteria, ensuring a civilian-led team is dispatched when appropriate (CityNews Staff, 2021; Reach Out Response Network, 2020).

The literature reveals a lack of clear, consistent definitions for the terms ‘non-emergency’ and ‘non-violent’. Two sources (Beck & Stagoff-Belfort, 2022; Provincial System Support Program & Makwa, 2023) discuss the need to better clarify what situations fall within and outside these domains. Provincial System Support Program & Makwa (2023) explain “a particular challenge has been establishing a clear understanding of the violence threshold for when to involve (or re-involve) 911 and police in Toronto Community Crisis Service” (p. 38), while (Beck & Stagoff-Belfort, 2022) notes a need to “identify the types of calls that are appropriate for civilian crisis response beyond those narrowly defined as behavioral health crises” (p. 5). Mellins (2021) explains that the Person in Crisis team in Rochester has been unreliable and somewhat ineffective, particularly due to the “strict criteria limiting who it can assist” (p. 2). Specifically, the Person in Crisis team does not respond to individuals under the influence of substances, which significantly limits who can access support from this team (Mellins, 2021).

### Theme 3: Team Uptake and Sustainability

Thirty of the articles included in this scoping review discussed the uptake and sustainability of civilian-led programs, emphasizing the importance of both initial interest and long-term support in restructuring crisis response models. The theme of *Team Uptake and Sustainability* highlights that social and political will for alternative crisis models, along with adequate resourcing, are critical factors that enable the development, implementation, and long-term viability of civilian-led crisis response teams.

#### Sub-Theme 3.1: Social and Political Will

Twenty-eight of the included sources discussed the interplay between the social and political will to introduce alternative crisis response teams in shaping the uptake and long-term sustainability of civilian-led crisis programs. Social will refers to the support and perceptions of community (Chalabi, 2019), while political will pertains to the commitment of elected officials and government bodies (Post et al., 2010). This relationship was discussed within the literature as reciprocal, with community advocacy influencing political action, and political decisions, in turn, shaping public engagement. In areas that have established civilian-led teams, support from local governments was a common trend in solidifying plans to establish alternative crisis response models (e.g., Fleming, 2021; Gorman, 2024; Townsend et al., 2023). Beck & Stagoff-Belfort (2022), Chrastil (2023), and Fleming (2021) highlight the impact of public perception and support in driving efforts to reimagine crisis response programs, acting as a push to inspire political will. Additionally, the support or "buy-in" from health professionals and law enforcement was identified as crucial in promoting civilian-led teams, particularly in championing their safety and effectiveness to local officials (Fleming, 2021; Gorman, 2024; Nonko, 2020). Local Progress Impact Lab (2022) notes that “as public interest in alternative responses has increased, so too has interest by local elected officials” (p. 5), while Fleming (2021) explains, “if not initially championed by community advocates, local officials may face pushback from community members” (p. 2).

#### Sub-Theme 3.2: Resourcing

Thirty-five articles discussed factors relating to the resourcing of civilian-led programs in contributing to their implementation and ongoing operation sustainability. Specifically, the literature spoke to partnership considerations and the provision of funding to support, operate, and uphold civilian-led teams. The importance of creating partnerships and collaborating with interest holders to support the operation of civilian-led services was discussed across the literature, particularly regarding the role of cross-sectoral, interprofessional collaboration (i.e., coordination between community organizations, healthcare services, government agencies, advocacy groups), in securing funding to sustain these teams (Mental Health Weekly, 2022b; Ryan, 2024; van Lier, 2021, 2022; Wellborn, 1999; Winters et al., 2015).

While the literature consistently emphasizes the need for sustained funding to support civilian-led programs, there are varying perspectives regarding the source and allocation of this funding. Some sources indicate that the funding of civilian-led programs should come directly from cuts to policing budgets (e.g., Towles, 2021a, 2021b; van Lier, 2021, 2022), such that these teams can be sustainably funded without relying on short-term grants or pilot project funding. Irwin and Pearl (2020) emphasize that areas looking to implement civilian-led teams should aim to provide a dedicated funding stream to sustain these teams, while recognizing that “local leaders may need to explore private funding sources to support the early implementation of the program, especially as governments are facing pandemic-related budget deficits” (p. 15). Alternatively, others (e.g., Egan-Elliott, 2021; Wellborn, 1999) argue that civilian-led programs should be “adding to the system, rather than cutting the police department’s budget” (Egan-Elliott, 2021, p. 4).

Mellins (2021) discusses three incidents of police violence in Rochester: a) the arrest and pepper-spraying of a 9-year-old girl in crisis; b) the tackling and pepper-spraying of a woman with her 3-year-old child following an alleged shoplifting incident; and c) and the fatal shooting of Tyshon Jones, a 29-year-old man in distress, who was threatening self-harm with a knife outside a homeless shelter. All three individuals were Black. These incidents occurred despite the implementation and operation of Rochester’s Person in Crisis team, a civilian-led crisis response model. Mellins (2021) notes, “that these incidents occurred after the [Person in Crisis] team was announced demonstrates that the team is either underfunded and therefore ill-equipped, or intentionally toothless and ill-conceived” (p. 2). Without proper resourcing, civilian-led teams risk being rendered futile.

## Discussion

The findings from this scoping review demonstrate the key processes involved in the restructuring of crisis response models into civilian-led teams. This review provides a synthesis of these key processes and can serve as a roadmap for communities interested in establishing civilian-led crisis response teams. Further, our findings illustrate how civilian-led models begin to recognize and respond to sanist, racist, and settler colonial underpinnings of policing mental health. Within the context of crisis response, transformative change seeks to support gradual shifts toward more community-based and non-carceral interventions (Ritchie, 2023). Accordingly, excluding police from staffing of crisis response teams is positioned toward advancing transformative change. The influence of collective awareness in shifting crisis response away from police highlights the power of mobilized efforts in driving systems level change (Ritchie, 2023). The recognition of longstanding community advocacy, and the accompanied pressure on elected officials, facilitated the decision to decenter police and reflects a broader redistribution of power toward the community, aligning with the transformative ethos of *nothing about us without us that is central to peer support.* (Antojado, 2023).

Establishing a distinct team composition that removes police from staffing structures represents an important shift in how communities conceptualize and deliver crisis response. This approach acknowledges the disproportionate harms experienced by individuals in acute distress, particularly Black and Indigenous people, during encounters with police, and aims to develop crisis care rooted in health and community support. Excluding police from direct response provides an opportunity to question long-standing assumptions that distress necessarily entails violence or danger and therefore requires police involvement (Haag, 2022; Interrupting Criminalization et al., 2022). The inclusion of peers in civilian-led models marks a meaningful shift toward valuing lived experience in crisis care. It challenges dominant assumptions that equate mental illness with incapacity (Kara, 2014, as cited in Shore, 2015, p. 10), and aligns with the PTMF’s emphasis on understanding distress through the lens of personal experience and meaning-making (Johnstone & Boyle, 2018). In this view, peer knowledge is positioned as a vital and legitimate source of insight.

Two important considerations remain. First, although these teams do not staff police, police may still be involved as secondary or tertiary supports: “we can escalate [involve police] where needed” (Ryan, 2024, p. 2). Greater transparency is needed in describing when and how police are engaged to ensure that the limits of civilian-led responses are clearly understood. Second, the participation of regulated health professionals within these teams can reproduce aspects of traditional authority and control (Seltzer et al., 2024). Although distinct from law enforcement, mental health clinicians may still hold the authority to interpret distress through clinical and risk-management frameworks and, in some circumstances, initiate involuntary assessment or hospitalization due to statutory obligations and professional duties related to safety concerns (Jones & Jacobs, 2024).

In response, several civilian-led models emphasized consent-based approaches, peer involvement, and Indigenous- and Black-led service delivery as ways of reducing power differentials and avoiding the replacement of one coercive response with another (Chambers & Deshman, 2024; Provincial System Support Program & Makwa, 2023). However, even when labelled as consent-based, these teams may still involve police or emergency services in situations involving mandated reporting obligations or perceived safety concerns, highlighting ongoing tensions between voluntary care, risk management, and individual autonomy (Chambers & Deshman, 2024; Provincial System Support Program & Makwa, 2023; Jones & Jacobs, 2024; Ryan, 2024). These findings raise important considerations regarding how team composition may influence approaches to crisis intervention, risk assessment, and escalation practices. While removing police from frontline roles represents meaningful progress, it does not fully separate crisis response from the broader institutional structures that can constrain autonomy and choice in mental healthcare (Hon-Sing Wong, 2024). Future research may benefit from exploring whether varying levels of clinical, peer, and community involvement shape how civilian-led teams navigate tensions related to safety, autonomy, consent, and involuntary intervention in practice.

The operational framework of each team, including the dispatch logistics and criteria for a response, requires careful consideration. As recognized by some of the included literature (e.g., Townend et al., 2023), calling 911 does not introduce the same sense of supposed universal safety for all, given its ties to policing. To respond to this, as per the literature, some teams have created alternative avenues to access civilian-led teams such as texting lines (Tharp et al., 2024). Decisions relating to team contact point should always be made in close consultation with community members, to ensure that teams remain accessible for all (Reach Out Response Network, 2020). Further, the literature identified a series of consistent criteria for a civilian-led response (i.e., situations that a civilian-led team may respond to) including the keywords ‘non-violent’ and ‘non-emergency’. However, as demonstrated in our findings, no clear parameters defining what constitutes a non-violent or non-emergency situation have been set. Because perceptions of violence – and of who is considered violent – are socially constructed and context dependent (Burstow, 2005), and distress is often conflated with danger, the lack of clear definitions raises important concerns for practice (Johnstone & Boyle, 2018). Emergency services may also be less available for people with severe mental illness, depending on context (White et al., 2014). Clearer articulation and parameters of these terms are needed to ensure crisis response practices remain equitable and contextually informed.

The sustainability of civilian-led response models remains closely tied to social and political support for alternatives to police-led crisis intervention. While many civilian-led models have expanded over the past decade, their long-term viability remains vulnerable to shifting political priorities and funding landscapes. For example, recent policy decisions across Canada, including reductions in harm reduction services in Ontario and Saskatchewan (Bayoumi et al., 2024; Government of Saskatchewan, 2024a, 2024b) and continued underfunding of social and health services alongside major budget cuts in Nova Scotia (Government of Nova Scotia, 2026), illustrate how broader political and economic contexts shape the resources available for transformative, community-based interventions (Brown et al., 2022; Ritchie, 2023). As such, continued advocacy from local elected leaders, community members, healthcare professionals, and organizers remains critical to sustaining and advancing civilian-led crisis response models.

### Implications for Policy and Practice

This scoping review of 46 sources provides an overview of the key processes involved in restructuring crisis response programs into civilian-led teams. Our findings demonstrate consistent overarching themes and associated subthemes involved in this restructuring and provide insight into how these teams have begun to respond to the legacies of sanism and racism within mental healthcare systems. We suggest areas looking to establish civilian-led crisis response teams consider the pathways to restructuring presented here.

A consistent implementation challenge involved the sustainability of funding for civilian-led crisis teams. While sources differed in their perspectives regarding where funding should originate (e.g., Ryan, 2024; van Lier, 2021, 2022; Wyton, 2022), the literature consistently emphasized the need for stable, ongoing investment rather than short-term or pilot-based funding arrangements (e.g., Towles, 2021a, 2021b; van Lier, 2021, 2022). An important policy implication emerging from this review is the need to embed civilian-led crisis response within sustained systems-level funding structures to support long-term program continuity and implementation (Ryan, 2024; Towles, 2021a, 2021b; van Lier, 2021, 2022; Wyton, 2022). At the practice level, we recommend civilian-led teams establish transparent, clear guidelines about their ties to police (i.e., when police will be involved, what their relationship to police looks like), as well as look to better define what constitutes ‘non-emergency’ or ‘non-violent’ situations, ensuring that human distress is not being conflated with violence.

### Limitations

A systematic review and meta-analysis were not possible due to the limited availability of empirical research on civilian-led crisis teams (Cumpston et al., 2019). Restricting the review to English reports limited the search scope. Due to the diverse language used to describe civilian-led teams, some articles may have been missed despite our multifaceted search strategy. In addition, we did not include anti-carceral crisis lines (e.g., Walls Down Collective, Trans Lifeline) as these services typically do not offer in-person intervention, and therefore did not meet our eligibility criteria for a civilian-led crisis response team.

### Areas for Future Research

Future research programs can explore the comparative effectiveness (i.e., service user experiences, linkage to resources), operational dynamics (e.g., number of calls received/responded to, time from call to response, length of intervention), and community perceptions between co-responder and civilian-led crisis response teams. Examining how these teams interact, differ in their approaches, and influence crisis outcomes could provide valuable insights into the strengths and limitations of each model, as well as inform best practices for scaling civilian-led crisis response. Further, exploring entirely anti-carceral crisis response teams (i.e., ones that have a strict, transparent policy against involving police) remains an important area for future research. Anti-carceral, peer-led care work offers insight into crisis intervention strategies that do not rely on law enforcement at any stage, demonstrating that entirely anti-carceral care is possible.

## Conclusion

The growing recognition of the harms of policing mental health within mainstream discourse has generated calls for alternative, non-police crisis response programs in form of civilian-led teams. This review provides a synthesis of the existing literature on civilian-led crisis teams and outlines the overarching themes associated with restructuring crisis models into civilian-led teams. It also examines how these teams have begun responding to the legacies of sanism, racism, and colonialism in policing mental health. In sum, this review brings together existing literature on civilian-led models, providing a foundation by which local leaders, policymakers, and communities can build off in exploring civilian-led crisis models.

## Data Availability

We have uploaded publicly available data derived from this review to Borealis: https://doi.org/10.5683/SP3/OXKHUZ

## Declarations

### Guideline statement

This scoping review was reported in accordance with the PRISMA (Preferred Reporting Items for Systematic Reviews and Meta-Analyses) reporting guidelines.

## References

[CR1] Anene, E., Nallajerla, M., Bath, E. P. J., & Castillo, E. G. (2023). Revisiting research safety protocols: The urgency for alternatives to law enforcement in crisis intervention. *Psychiatric Services,**74*(3), 325–328. 10.1176/appi.ps.20220084 36004437 PMC10027434

[CR2] Antojado, D. (2023). “Nothing about us without us”: Analyzing the potential contributions of lived experience to penological pedagogy. *Journal of Criminal Justice Education*. 10.1080/10511253.2023.2275101

[CR3] Badwall, H. (2022). Critical race theory for social work. In S. S. Shaikh, B. A. LeFrancois, & T. Macias (Eds.), *Critical social work praxis* (pp. 245–263). Fernwood Publishing.

[CR4] Bayoumi, A., Wu, M., Pogacar, F., Wang, T., & Gomes, T. (2024). *Estimating the effects of closing supervised consumption sites in Toronto: Use of services*. MAP Centre for Urban Health Solutions & Ontario Drug Policy Research Network. https://odprn.ca/wp-content/uploads/2024/11/Estimating-the-Effects-of-Closing-Supervised-Consumption-Sites-in-Toronto.pdf

[CR5] Beck, J., Stagoff-Belfort, A., & Tan de Bibiana, J. (2022). *Civilian Crisis Response: A Toolkit for Equitable Alternatives to Police*. https://www.vera.org/civilian-crisis-response-toolkit

[CR6] Benjamin, A. L. (2003). *The Black/Jamaican criminal: The making of ideology*.[Doctoral dissertation, University of Toronto]. ProQuest Dissertations Publishing.

[CR7] Black, C., & Calhoun, A. (2022). How biased and carceral responses to persons with mental illness in acute medical care settings constitute iatrogenic harms. *AMA Journal of Ethics,**24*(8), 781–787. 10.1001/amajethics.2022.781 35976936

[CR8] Brown, C., Johnstone, M., Ross, N., & Doll, K. (2022). Challenging the constraints of neoliberalism and biomedicalism: Repositioning social work in mental health. *Qualitative Health Research,**32*(5), 771–787. 10.1177/10497323211069681 35382646

[CR9] Burstow, B. (2005). A critique of posttraumatic stress disorder and the DSM. *Journal of Humanistic Psychology,**45*(4), 429–445. 10.1177/0022167805280265

[CR10] Campbell, D. A. (2023). *2023 Update on the Toronto Community Crisis Service and Proposed Expansion Plan*. City of Toronto Social Development, Finance & Administration Division. https://www.toronto.ca/wp-content/uploads/2023/10/90a4-2023-Update-on-the-Toronto-Community-Crisis-Service-and-Proposed-Expansion-Plan.pdf

[CR11] Canady, V. A. (2021). Emerging 988 crisis hotline expected to transform BH care. *Mental Health Weekly,**31*(21), 3–5. 10.1002/mhw.32810

[CR12] Chalabi, A. (2019, March 6). *“Social will” as important as political will for implementing human rights*. Oxford Human Rights Hub. https://ohrh.law.ox.ac.uk/social-will-as-important-as-political-will-for-implementing-human-rights/

[CR13] Chambers, J., & Deshman, A. (2024, June 18). Alternatives to police during responses to mental health crisis in Toronto. *International Network of Civil Liberties Organizations*. https://inclo.net/pillars/civic-space/in-our-hands/alternatives-to-police-during-responses-to-mental-health-crisis-in-toronto/

[CR14] Chrastil, N. (2023, June 5). *City of New Orleans rolls out non-police 911 mental health response.* The Louisiana Weekly. https://www.louisianaweekly.com/city-of-new-orleans-rolls-out-non-police-911-mental-health-response/

[CR15] CityNews Staff. (2021, June 24). *Toronto police pilot program will see mental health calls diverted to crisis centre*. City News*.* https://toronto.citynews.ca/2021/06/24/toronto-police-pilot-program-will-see-mental-health-calls-diverted-to-crisis-centre/

[CR16] Cotton, D., & Coleman, T. G. (2010). Canadian police agencies and their interactions with persons with a mental illness: A systems approach. *Police Practice and Research,**11*(4), 301–314. 10.1080/15614261003701665

[CR17] CSG Justice Center. (2021, December). *Overview of First Responder Programs.* https://csgjusticecenter.org/publications/expanding-first-response/the-toolkit/

[CR18] Cumpston, M., Li, T., Page, M. J., Chandler, J., Welch, V. A., Higgins, J. P., & Thomas, J. (2019). Updated guidance for trusted systematic reviews: A new edition of the Cochrane handbook for systematic reviews of interventions. *The Cochrane Database of Systematic Reviews*. 10.1002/14651858.ED000142 31643080 PMC10284251

[CR19] DeLaus, M. (2020, November 16). *Alternatives to police as first responders: Crisis response programs*. Government Law Center, Albany Law School. https://www.albanylaw.edu/government-law-center/alternatives-police-first-responders-crisis-response-programs

[CR20] Eaton, A. D., Rowe, M., Nguyen, A., Lowe, L., Fletcher, K., Adenwala, A., Sudom-Young, S., Rackow, R., Poellet, L., McDonald, M., & McInroy, L. B. (2025). Processes of co-response crisis mental health models: A scoping review. *Advances in Mental Health*. 10.1080/18387357.2025.2522434

[CR21] Egan-Elliott, R. (2021, October 8). Pilot project would see non-police responders sent to mental-health crises. *Victoria Times Colonist*. https://www.timescolonist.com/local-news/pilot-project-would-see-non-police-response-sent-to-mental-health-crisis.

[CR22] Fagan, M. J., Wunderlich, K. B., & Faulkner, G. (2025). Psychological distress among Canadian postsecondary students: A repeated cross-sectional analysis of the Canadian campus wellbeing survey (CCWS) between spring 2020–2023. *Journal of American College Health,**73*(8), 3201–3206. 10.1080/07448481.2024.2382438 39083793

[CR23] Fleming, E. (2021). *Local legislation as a pathway to building community responder programs.* Justice Center: The Council of State Governments. https://csgjusticecenter.org/2021/09/07/local-legislation-as-a-pathway-to-building-community-responder-programs/

[CR24] Foucault, M. (1961). *Madness and civilization: A history of insanity in the age of reason*. Pantheon Books. 10.1192/bjp.bp.115.165274 26527668

[CR25] Geller, J. L., Fisher, W. H., & McDermeit, M. (1995). A national survey of mobile crisis services and their evaluation. *Psychiatric Services,**46*(9), 893–897. 10.1176/ps.46.9.893 7583498

[CR26] Gillis, W. (2023). These mental health advocates are working on an alternative to police intervention when someone is in crisis. They say ‘all of the sudden’ people are interested. *The Toronto Star.* https://www.thestar.com/news/gta/these-mental-health-advocates-are-working-on-an-alternative-to-police-intervention-when-someone-is/article_9e53dfe1-944f-50e2-afd2-03e00c0888e1.html

[CR27] Goldman, M. L., Looper, P., & Odes, R. (2023). *National survey of mobile crisis teams: Summary report*. Vibrant Emotional Health.

[CR28] Gorman, M. (2024, May 16). *N.S. government working to launch mental health crisis response team*. CBC News*.* https://www.cbc.ca/news/canada/nova-scotia/mental-health-crisis-response-brian-comer-1.7206527

[CR29] Government of Nova Scotia. (2026, February 23). *Budget 2026–27: Defending Nova Scotia*. https://news.novascotia.ca/en/2026/02/23/budget-2026-27-defending-nova-scotia

[CR30] Government of Saskatchewan. (2024, January 18). *Province realigns health system approach to illicit drug use issues*. https://www.saskatchewan.ca/government/news-and-media/2024/january/18/province-realigns-health-system-approach-to-illicit-drug-use-issues

[CR31] Government of Saskatchewan. (2024, September 5). *Government extends funding for regina street team outreach program*. https://www.saskatchewan.ca/government/news-and-media/2024/september/05/government-extends-funding-for-regina-street-team-outreach-program

[CR32] Haag, J. (2022). Police use of force in Canada: Dispelling the myth of difference. In A. Stadnyk, K. Walby, & S. Pasternak (Eds.), *Disarm, Defund Dismantle Police Abolition in Canada. *Between the Lines.

[CR33] Harvey, D. (2005). *A brief history of neoliberalism*. Oxford University Press.

[CR34] Herson, K. (2018). *Mad minds: Theorizing the intersection of gender, sexuality, and mental illness in contemporary media discourse* [Doctoral dissertation, Arizona State University]. ASU Library.

[CR35] Hilal, S. M., & Jones, D. S. (2014). A package deal: Police, fire, and EMS all in one. *Police Chief Magazine*. https://www.policechiefmagazine.org/a-package-deal-police-fire-and-ems/

[CR36] Hon-Sing Wong, E. (2016). The brains of a nation: The eugenicist roots of Canada’s mental health field and the building of a white non-disabled nation. *Canadian Review of Social Policy,**75*(75), 1–29.

[CR37] Hon-Sing Wong, E. (2024). Mental health workers have never been the solution to racial violence by police. In C. Fortier, E. Hon-Sing Wong, & M. Rwigema (Eds.), *Abolish Social Work (As We Know It)* (pp. 32–43). Between the Lines.

[CR38] Hon-Sing Wong, E., Rwigema, M. J., Penak, N., & Fortier, C. (2022). Abolishing carceral social work. In S. Pasternak, K. Walby, & A. Stadnyk (Eds.), *Disarm. Defund. Dismantle: Police abolition in Canada* (pp. 145–153). Between the Lines.

[CR39] Hong, Q. N., Fàbregues, S., Bartlett, G., Boardman, F., Cargo, M., Dagenais, P., Gagnon, M.-P., Griffiths, F., Nicolau, B., O’Cathain, A., Rousseau, M.-C., Vedel, I., & Pluye, P. (2018). The Mixed Methods Appraisal Tool (MMAT) version 2018 for information professionals and researchers. *Education for Information,**34*(4), 285–291. 10.3233/EFI-180221

[CR40] Hoult, J. (1996). Mobile crisis services. *Psychiatric Services,**47*(3), 309–310. 8820561 10.1176/ps.47.3.309b

[CR41] Iacobucci, F. (2014). *Police Encounters with People in Crisis*. Toronto Police Service.

[CR42] Interrupting Criminalization, Project Nia, and Critical Resistance. (2022). *So is this Actually an Abolitionist Proposal or Strategy?.* Critical Resistance. https://criticalresistance.org/resources/actually-an-abolitionist-strategy-binder/

[CR43] Irwin, A., & Pearl, B. (2020, October 28*). The community responder model: How cities can send the right responder to every 911 call*. Center for American Progress. https://www.americanprogress.org/article/community-responder-model/

[CR44] Jackson, J., & Bradford, B. (2019). *Measuring public attitudes towards the police*. Public Safety Canada.

[CR45] Johnstone, L., & Boyle, M. (2018). *The Power threat meaning framework: Towards the identification of patterns in emotional distress, unusual experiences and troubled or troubling behaviour, as an alternative to functional psychiatric diagnosis*. British Psychological Society.

[CR46] Jones, N., & Jacobs, L. A. (2024). Abolitionist and harm reduction praxis for public sector mental health services: An application to involuntary hospitalization. In C. Fortier, E. Hon-Sing Wong, & M. Rwigema (Eds.), *Abolish social work (as we know it)* (pp. 196–215). Between the Lines.

[CR47] Justice Action (2023). *Crisis intervention support.* https://justiceaction.org.au/wp-content/uploads/2023/08/Crisis-Intervention-Support-.pdf

[CR48] Kara, F. B. (2014). Police interactions with the mentally ill: The role of procedural justice. *Canadian Graduate Journal of Sociology and Criminology,**3*(1), 79–84.

[CR49] Karlsson, B., Borg, M., Biong, S., Ness, O., & Kim, H. S. (2012). A crisis resolution and home treatment team in Norway: A longitudinal survey study part 2. Provision of professional services. *International Journal of Mental Health Systems,**6*(1), 14. 22958549 10.1186/1752-4458-6-14 PMC3487748

[CR50] Kearnes, L. C., (2022). *Calgary Community Mobile Crisis Response Pilot Project Overview.* City of Calgary. https://www.calgary.ca/content/dam/www/csps.cns/document

[CR51] Koziarski, J. (2018). *Policing mental health: An exploratory study of crisis intervention teams and co-response teams in the Canadian context (1380435*) [Master’s thesis, University of Ontario Institute of Technology]. ProQuest Dissertation Publishing.

[CR52] Lee, J. (2013). Mad as hell: The objectifying experience of symbolic violence. In B. Lefrancois, R. Menzies, & G. Reaume (Eds.), *Mad matters: A critical reader in Canadian mad studies* (pp. 105–121). Canadian Scholars.

[CR53] Legal Defense Fund., & Bazelon Center. (2022, July). *Advancing an alternative to police: Community-based services for Black people with mental illness.* https://www.naacpldf.org/wp-content/uploads/2022.07.06-LDF-Bazelon-Brief-re-Alternative-to-Policing-Black-People-with-Mental-Illness.pdf

[CR54] Liegghio, M. (2013). A denial of being: Psychiatrization as epistemic violence. In B. Lefrancois, R. Menzies, & G. Reaume (Eds.), *Mad matters: A critical reader in Canadian mad studies* (pp. 105–121). Canadian Scholars.

[CR55] Livingston, J., & Chambers, J. (2024). Civilian mobile crisis services. In A. Szigeti, R. Dhand, D. Bonnet, & J. Presser. (Eds.), *Canadian Anthology on Mental Health and Law.*

[CR56] Local Progress Impact Lab. (2022). *Reform/transform: Creating a community responder program.* https://localprogress.org/wp-content/uploads/2022/10/Reform-Transform-Creating-a-Community-Responder-Program.pdf

[CR57] Lowder, E. M., Grommon, E., Bailey, K., & Ray, B. (2024). Police-mental health co-response versus police-as-usual response to behavioral health emergencies: A pragmatic randomized effectiveness trial. *Social Science & Medicine*. 10.1016/j.socscimed.2024.116723 38422686

[CR58] Marcoux, J., D. & Nicholson, K. (2018, April 5). *Deadly force*. CBC News. https://newsinteractives.cbc.ca/longform-custom/deadly-force

[CR59] Marcus, N., & Stergiopoulos, V. (2022). Re-examining mental health crisis intervention: A rapid review comparing outcomes across police, co-responder and non-police models. *Health & Social Care in the Community,**30*(5), 1665–1679. 10.1111/hsc.13731 35103364

[CR60] Maynard, R. (2017). *Policing Black lives: State violence in Canada from slavery to the present*. Fernwood Publishing.

[CR61] Meerai, S., Abdillahi, I., & Poole, J. (2016). An introduction to anti-Black sanism. *Intersectionalities: A Global Journal of Social Work Analysis, Research, Polity and Practice,**5*(3), 18–35.

[CR62] Mellins, S. (2021, April 28). *In Rochester, a police alternative delivers…police*. In These Times. https://inthesetimes.com/article/replacing-police-pic-rochester-crisis-response.

[CR63] Mental Health Commission of Canada. (2025). *Peer Support*. https://mentalhealthcommission.ca/what-we-do/access/peer-support/

[CR64] Mental Health Weekly. (2022a). New Hampshire crisis units offer new tool in MH treatment. *Mental Health Weekly,**32*, 7–8. 10.1002/mhw.33080

[CR65] Mental Health Weekly. (2022b). San Diego officials tout success of mobile crisis teams to treat mental health and drug abuse. *Mental Health Weekly,**32*(10), 8. 10.1002/mhw

[CR66] Mukherjee, A. (2022). Systematic review or scoping review? Guidance for authors when choosing between a systematic or scoping review approach. *BMC Medical Research Methodology,**18*(1), 143. 10.1186/s12874-018-0611-x PMC6245623 30453902

[CR67] Munn, Z., Peters, M. D. J., Stern, C., Tufanaru, C., McArthur, A., & Aromataris, E. (2018). Systematic review or scoping review? Guidance for authors when choosing between a systematic or scoping review approach. *BMC Medical Research Methodology,**18*(1), 143. 10.1186/s12874-018-0611-x 30453902 PMC6245623

[CR68] Ngabo, G. (2021, March 19). Toronto balcony fall demonstrates need for non-police first-responders, mental health experts say. *The Star*. https://www.thestar.com/news.gta/toronto-balcony-fall-demonstrates-thr-need-for-non-police-respnders-mental-health-experts-say/article-287674a0-a7do-5754-9e71-68b3052b459d-html

[CR69] Nishar, S., Jaiswal, J., Mandal, S., Brumfield, E., & Soske, J. (2025). They judge you by your incarceration: A qualitative study of mistrust among formerly incarcerated people navigating mental health care. *Administration and Policy in Mental Health and Mental Health Services Research,**52*(3), 561–569. 10.1007/s10488-025-01435-0 40234348

[CR70] Nonko, E. (2020, Oct 1). *A volunteer-run program could model for mental health responses without police intervention*. Next City. https://nextcity.org/urbanist-news/volunteer-run-program-model-mental-health-response-police-intervention

[CR71] Page, C., & Woodland, E. (2023). *Healing justice lineages: Dreaming at the crossroads of liberation, collective care, and safety*. Findaway World.

[CR72] Pepler, E. F., & Barber, C. G. (2021). Mental health and policing: Picking up the pieces in a broken system. *Healthcare Management Forum,**34*(2), 93–99. 10.1177/0840470420979635 33499683

[CR73] Pham, M. T., Rajić, A., Greig, J. D., Sargeant, J. M., Papadopoulos, A., & McEwen, S. A. (2014). A scoping review of scoping reviews: Advancing the approach and enhancing the consistency. *Research Synthesis Methods,**5*(4), 371–385. 10.1002/jrsm.1123 26052958 PMC4491356

[CR74] Poole, J. M., Jivraj, T., Arslanian, A., Bellows, K., Chiasson, S., Hakimy, H., & Reid, J. (2012). Sanism, ‘mental health’, and social work/education: A review and call to action. *Intersectionalities: A Global Journal of Social Work Analysis, Research, Polity, and Practice,**1*, 20–36.

[CR75] Pope, L. G., Stagoff-Belfort, A., Warnock, A., de Bibiana, J. T., Watson, A. C., Wood, J., & Compton, M. T. (2023). Competing concerns in efforts to reduce criminal legal contact among people with serious mental illnesses: Findings from a multi-city study on misdemeanor arrests. *Administration and Policy in Mental Health and Mental Health Services Research,**50*(3), 476–487. 10.1007/s10488-023-01252-3 36717527 PMC9886548

[CR76] Post, L. A., Raile, A. N. W., & Raile, E. D. (2010). Defining political will. *Politics and Policy,**38*(4), 653–676. 10.1111/j.1747-1346.2010.00253.x

[CR77] Provincial System Support Program & Makwa, S. (2023). *Toronto Community Crisis Service: six-month implementation evaluation report*. Centre for Addictions and Mental Health. https://www.toronto.ca/wp-content/uploads/2023/03/9034-APPENDIXEToronto-Community-Crisis-Service-Six-Month-Implementation-Evaluation-Report.pdf

[CR78] Reach Out Response Network. (2020, November). Final Report Summary on Alternative Crisis Response Models for Toronto. https://csgjusticecenter.org/wp-content/uploads/2021/12/Summary-FinalReportonAlternativeCrisisResponseModelsforToronto.pdf

[CR79] Redgate, S., Clibbens, N., Haighton, C., Dalkin, S., Bate, A., Girling, M., McCarthy, S., Eagles, T., Gray, J., & McKinnon, I. (2025). Mechanisms to support interventions involving the police when responding to persons experiencing a mental health crisis: A realist review. *Health & Social Care in the Community*. 10.1155/hsc/7445445

[CR80] Ritchie, A. (2023). *Practicing new worlds*. AK Press.

[CR81] Roennfeldt, H., Hill, N., Byrne, L., & Hamilton, B. (2024). Exploring the lived experience of receiving mental health crisis care at emergency departments, crisis phone lines and crisis care alternatives. *Health Expectations: An International Journal of Public Participation in Health Care and Health Policy*. 10.1111/hex.14045 38590099 PMC11002315

[CR82] Rogers, M. S., McNiel, D. E., & Binder, R. L. (2019). Effectiveness of police crisis intervention training programs. *The Journal of the American Academy of Psychiatry and the Law,**47*(4), 414–421. 31551327 10.29158/JAAPL.003863-19

[CR83] Rosenbaum, N. (2010). Street-level psychiatry - A psychiatrist’s role with the Albuquerque Police Department’s Crisis Outreach and Support Team. *Journal of Police Crisis Negotiations,**10*(1–2), 175–181. 10.1080/15332581003757040

[CR84] Rowe, M., & Eaton, A. D. (2025). Data for restructuring crisis responde programs as civilian-led: A scoping review. *Borealis*. 10.5683/SP3/OXKHUZ PMC13588907 42366288

[CR85] Ryan, H. (2024, February 29). *Halifax council lays out plan to move away from police-led wellness checks.* CBC News. https://www.cbc.ca/news/canada/nova-scotia/detasking-police-halifax-plans-for-civilian-crisis-response-1.7130341

[CR86] Ryder, C., & Nash, A. (2024). *Civilian Crisis Response Rather than Co-Responder and CIT Programs*. Montgomery County Maryland. https://www.montgomerycountymd.gov/COUNCIL/Resources/Files/agenda/col/2024/20240116/testimony/item14-ClaireRyder.pdf

[CR87] Seltzer, C., Palmer, L., & Shahidi, G. (2024). Not criminally responsible. In C. Fortier, E. Hon-Sing Wong, & M. Rwigema (Eds.), *Abolish Social Work (As We Know It)* (pp. 70–81). Between the Lines.

[CR88] Shadravan, S. M., Edwards, M. L., & Vinson, S. Y. (2021). Dying at the intersections: Police-involved killings of black people with mental illness. *Psychiatric Services,**72*(6), 623–625. 10.1176/appi.ps.202000942 34110254

[CR89] Shimrat, I. (2013). The tragic farce of “community mental health care.” In B. Lefrancois, R. Menzies, & G. Reaume (Eds.), *Mad matters: A critical reader in Canadian mad studies* (pp. 144–157). Canadian Scholars.

[CR90] Shore, L, K. (2015). *Exploring interactions between police and people with mental illness (1761)* [Master’s thesis, Wilfrid Laurier University]. Scholars Commons @ Laurier.

[CR91] Simpson, L. B. (2017). The attempted dispossession of Kwe. In L. Betasamosake Simpson (Ed.), *As we have always done: Indigenous freedom through radical resistance* (pp. 39–54). University of Minnesota Press.

[CR92] Singh, I. (2020, July 23). *2020 already a particularly deadly year for people killed in police encounters, CBC research shows*. CBC News. https://newsinteractives.cbc.ca/features/2020/fatalpoliceencounters/

[CR93] Spolum, M., Lopez, W. D., Watkins, D. C., & Fleming, P. J. (2023). Police violence: Reducing the harms of policing through public health-informed alternative response program. *American Journal of Public Health*. 10.2105/AJPH.2022.307107 36696619 PMC9877383

[CR94] Taheri, S. A. (2016). Do crisis intervention teams reduce arrest and improve officer safety? A systematic review and meta-analysis. *Criminal Justice Policy Review,**27*(1), 76–96. 10.1177/0887403414556289

[CR95] Tharp, D., Kious, B. M., Bakian, A., Brewer, S., Langenecker, S., Schreiner, M., Shabalin, A., Coon, H., Welsh, R. C., & Medina, R. M. (2024). Assessing access: Texting hotline app provides mental health crisis care for economically deprived youth. *Social Science & Medicine*. 10.1016/j.socscimed.2024.117369 PMC11772012 39369499

[CR96] Thompson, L. K., Sugg, M. M., & Runkle, J. R. (2018). Adolescents in crisis: A geographic exploration of help-seeking behavior using data from crisis text line. *Social Science & Medicine,**215*, 69–79. 10.1016/j.socscimed.2018.08.025 30216891

[CR97] Towles, A. G. (2021a). *Three ways state leaders can support community responder programs.* Justice Center: The Council of State Governments. https://csgjusticecenter.org/2021/09/07/three-ways-state-leaders-can-support-community-responder-programs/

[CR98] Towles, A. G. (2021b). *Explainer: A Breakdown of Community Responder program staff models and structures*. CSG Justice Center. https://csgjusticecenter.org/2021/09/07/explainer-a-breakdown-of-community-responder-program-staff-models-and-structures/

[CR99] Townsend, T. G., Dillard-Wright, J., Prestwich, K., Alapatt, V., Kouame, G., Kubicki, J. M., Johnson, K. F., & Williams, C. D. (2023). Public safety redefined: Mitigating trauma by centering the community in community mental health. *American Psychologist,**78*(2), 227–243. 10.1037/amp0001081 37011172

[CR100] Tyndall, J. (2010). Grey literature in the health sciences: Evaluating grey lit. *Wright State University.* https://guides.libraries.wright.edu/c.php?g=978368&p=7080366

[CR101] van Lier, P. (2021, July 21). *Reimagining public safety in Cleveland*. Policy Matters Ohio. https://www.policymattersohio.org/research-policy/quality-ohio/justice-reform/reimagining-public-safety-in-cleveland

[CR102] van Lier, P. (2022). *Creating a care response model in Cleveland for those in crisis*. Policy Matters Ohio. https://policymattersohio.org/research/creating-a-care-response-model-in-cleveland-for-those-in-crisis/#:~:text=Care

[CR103] Walby, K. (2022). Against the social harms of policing. In S. Pasternak, K. Walby, & A. Stadnyk (Eds.), *Disarm, Defund, Dismantle: Police abolition in Canada* (pp. 44–51). Between the Lines.

[CR104] Watson, A. C., & El-Sabawi, T. (2023). Expansion of the police role in responding to mental health crises over the past fifty years: Driving factors, race inequities and the need to rebalance roles. *Law and Contemporary Problems,**86*(1), 1–28.

[CR105] Wellborn, J (1999). Responding to individuals with mental illness. *FBI Law Enforcement Bulletin*, *68*(11), 6–8. https://www.thefreelibrary.com/Responding-to-individuals-with-mental-illness-a058177901.

[CR106] White, J., Gutacker, N., Jacobs, R., & Mason, A. (2014). Hospital admissions for severe mental illness in England: Changes in equity of utilization at the small area level between 2006 and 2010. *Social Science & Medicine,**120*, 243–251. 10.1016/j.socscimed.2014.09.036 25262312 PMC4225455

[CR107] Winters, S., Magalhaes, L., & Kinsella, E. A. (2015). Interprofessional collaboration in mental health crisis response systems: A scoping review. *Disability and Rehabilitation,**37*(23), 2212–2224. 10.3109/09638288.2014.1002576 25586793

[CR108] Wyton, M. (2022, Nov 25). *A new model for responding to mental health crises*. The Tyee. https://thetyee.ca/News/2022/11/24/New-Model-Responding-Mental-Health-Crises/

[CR109] Yee, K. (2022). *Indigenous resurgence in colonial urban parks: Possibilities and potential for urban Indigenous land-based practices* [Master’s thesis, University of Toronto]. TSpace.

[CR110] Yousif, N. (2020, September 17). Toronto moves forward on consultations to create a non-police mental health crisis response team*. Toronto Star,* https://www.thestar.com/news/gta/toronto-moves-forward-on-consultations-to-create-a-non-police-mental-health-crisis-response-team/article_a69b69ca-7bc2-5ed2-a8e3-9cb6aefac021.html

[CR111] Yousif, N. (2022a, January). Toronto’s first-ever mental healthcrisis response teams — without police— to launch in March. *Toronto Star,* https://www.thestar.com/news/gta/toronto-moves-forward-on-consultations-to-create-a-non-police-mental-health-crisis-response-team/article_a69b69ca-7bc2-5ed2-a8e3-9cb6aefac021.html

[CR112] Yousif, N. (2022b, October 17). Toronto approved a non-police crisis response team. This woman is trying to build them. *Toronto Star,* https://www.thestar.com/news/gta/toronto-approved-non-police-crisis-response-teams-this-woman-is-trying-to-build-them/article_5cbd7130-c4f9-5bf7-b4f1-cb067caad97d.html

[CR113] Zealberg, J. J., Santos, A. B., & Fisher, R. K. (1993). Benefits of mobile crisis programs. *Hospital & Community Psychiatry,**44*(1), 16–17. 10.1176/ps.44.1.16 8436356

[CR114] Ziafati, N. (2022, March 24). ‘*Time for change’: Toronto launching services to respond to mental health crisis*. The Canadian Press. https://www.ctvnews.ca/toronto/article/time-for-change-toronto-launching-service-to-respond-to-mental-health-crisis-calls/

